# Identification and validation of cuproptosis-related LncRNA signatures as a novel prognostic model for head and neck squamous cell cancer

**DOI:** 10.1186/s12935-022-02762-0

**Published:** 2022-11-11

**Authors:** Xiajing Liu, Wenwei Cheng, Heqing Li, Yexun Song

**Affiliations:** 1 Department of Otolaryngology-Head Neck Surgery, The Third Xiangya Hospital of Central South University, Changsha, 410013 Hunan Province China; 2 Graduate School of Guilin Medical University, Guilin, 541004 The Guangxi Zhuang Autonomous Region China; 3 The Third Xiangya Hospital of Central South University, Changsha, 410013 Hunan Province China; 4 Xiangya School of Public Health, Central South University, Changsha, 410000 Hunan Province China

**Keywords:** Cuproptosis, Head and neck squamous cell cancer, lncRNA, Prognosis, Biomarker

## Abstract

**Background:**

Head and neck squamous cell cancer (HNSCC) is a common malignant cancer. We aimed to explore prognostic cuproptosis-related lncRNAs (CRLs) and prognostic risk models for HNSCC.

**Methods:**

The transcriptome profiles and clinical data were obtained from the TCGA database, and 19-cuproptosis-related genes (CRGs) were acquired from previous studies. Then, the prognostic model based on seven CRLs was established. We analysed its value to evaluate the prognosis, drug sensitivity, and tumour immune functions of patients with HNSCC. Finally, we used quantitative reverse transcription polymerase chain reaction (qRT‒PCR) to validate the seven CRLs.

**Results:**

We established a 7-CRL signature. Kaplan‒Meier (K–M) curve analysis demonstrated a significantly preferable prognosis in the low-risk group. Multivariate Cox regression analysis revealed that the risk score could serve as an independent prognostic factor. Nomogram, ROC curve, and principal component analysis indicated that the signature presented significant predictive capability. Moreover, most of the high-risk group showed lower levels of IC_50_ for certain chemotherapy drugs, such as cisplatin, cytarabine, docetaxel, doxorubicin, etoposide, gemcitabine, methotrexate, paclitaxel, and dasatinib. Finally, the expression of AP001372.2, MIR9-3HG, AL160314.2, POLH-AS1, and AL109936.2 was upregulated, while AC090587.1 and WDFY3-AS2 were downregulated in HNSCC cell lines compared with normal cell lines by qRT‒PCR.

**Conclusions:**

The 7-CRL signature was presented to be a novel biomarker for predicting prognosis for HNSCC.

**Supplementary Information:**

The online version contains supplementary material available at 10.1186/s12935-022-02762-0.

## Background

Head and neck cancer (HNC) ranked sixth among all cancer types worldwide, which includes cancers originating in the lip, oral cavity, nasopharynx, larynx, hypopharynx, etc. [1]. According to the data based on the GLOBOCAN produced by the International Agency for Research on Cancer in 2020, the global incidence counts of HNC were approximately 870,000 and the global mortality counts of HNC were approximately 440,000 [2], which indicates that more attention should be paid on the significant burden caused by HNC.

Head and neck squamous cell cancer (HNSCC), as the most common pathological type of HNC, accounts for approximately 90% of HNC cases. During the past decades, although significant improvement has been observed in the treatment strategies including surgery, radiotherapy, chemotherapy, and immunotherapy for HNSCC, the overall survival and quality of life of HNSCC has not increased accordingly [3]. Therefore, it is urgently needed to further explore the development and progression mechanisms, screening effective biomarkers for predicting early diagnosis, and long-term prognosis of HNSCC.

As an important cofactor, copper plays a vital role in various physiological processes [4]. Dysregulated intracellular bioavailability of copper can lead to oxidative stress and cytotoxicity [5]. However, many associations between disease status and Cu have been observed. In particular, copper levels in the serum and tumour tissues of patients with various cancers were significantly changed [6–11]. In a recent study published in the journal Science, a novel form of cell death, copper-dependent cell death (referred to as cuproptosis), showed that copper binds directly to lipoylated components of the tricarboxylic acid (TCA) cycle, followed by lipoylated mitochondrial protein aggregation and subsequent loss of the Fe–S cluster, triggering proteotoxic stress and a unique form of cell death [12]. Therefore, the potential role and mechanism of cuproptosis in the development and progression of HNSCC needs to be further explored.

To the best of our knowledge, there were only 19 coding genes verified to be related to cuproptosis, which was not enough to establish a prognostic model for HNSCC. Previous studies suggested that lncRNAs have a potential role in the diagnosis and therapy of HNSCC [13]. At present, there are few studies focusing on CRLs in HNSCC, which are used to predict the prognosis and chemotherapy sensitivity of HNSCC. Therefore, we aimed to explore cuproptosis-associated lncRNAs, which might provide new ideas and insights for HNSCC treatment and prediction.

Based on seven CRLs, our study revealed a predictive signature model and evaluated the prognosis, drug sensitivity, and tumour immune functions of patients with HNSCC. Ultimately, we also tentatively verified the expression levels of seven CRLs among HNSCC cell lines and human normal nasopharyngeal cell lines.

## Materials and methods

### Data download and screening of cuproptosis-related lncRNAs

The transcriptome profiles and relevant clinical information of patients with HNSCC were downloaded from the TCGA database. Gene expression was normalized by the “limma” R package. A total of 19 CRGs were acquired from published studies (Additional file 1: Table S1).

The association between the CRLs and HNSCC was estimated by Pearson correlation. The correlation coefficient |*R*^2^|> 0.4 at *P* < 0.001 was deemed significant. A Sankey diagram was drawn to present the extent of association between CRLs and CRGs via the ‘ggalluvial’ R package.

### Establishment of the cuproptosis-related lncRNA prognostic model

Cox regression was performed by the “survival” and “glmnet” packages. Patients with HNSCC were randomly separated into training or test groups. We used the 783 CRLs identified to establish the HNSCC prognostic model. Then, prognostic candidates were identified by LASSO Cox regression analysis. Ultimately, we generated a prognostic model for seven lncRNAs associated with cuproptosis, selecting the best penalty parameter λ correlated with at least tenfold cross-validation. According to the median risk score, the samples were classified into a high-risk group and low-risk group. The K–M curve was generated by the ‘survminer’ R package. A receiver operating characteristic curve (ROC) was generated by the ‘timeROC’ R package.

### Construction and validation of a predictive nomogram

The nomogram of HNSCC patients was plotted by the “rms” package. The calibration curve was performed to evaluate the consistency between the OS and PFS rates in this study.

### Principal component analysis (PCA)

PCA is a helpful tool that is widely applied in reducing dimensionality and extracting features in the computer vision field [14]. We also estimated differences between the above two risk groups by the “scatterplot3d” package.

### Functional enrichment analysis

The genes differentially expressed between the high-risk and low-risk groups were identified (|log_2_(fold change)|> 1 and FDR < 0.05) by the ‘edgeR’ R package [15]. GO and KEGG analyses were conducted by the ‘clusterProfiler’ R package [16].

### Tumour mutation burden (TMB) analysis

TMB refers to the number of mutations per million bases in the tumour genome [17]. Tumours with a higher TMB were usually more sensitive to immunotherapy. The TMB was analysed by the “maftools” R package.

### Immune functions and tumour immune dysfunction and exclusion (TIDE) score

Based on the CRL signature, single-sample gene set enrichment analysis (ssGSEA) and the ‘‘gsva’’ package were compared to determine the immune functions between the high-risk and low-risk groups. The differences in immune functions were uncovered using a heatmap. The TIDE score was used for the prediction of outcome and response to immunotherapy for cancer patients. The TIDE score, CAF, IFN-g (IFNG), CD8 score, CD274 score, MDSC, dysfunction score, merck18 (T-cell-inflamed signature) score, TAM M2, and exclusion score were obtained from the TIDE web (http://tide.dfci.harvard.edu).

### Drug susceptibility analysis

The half-maximal inhibitory concentration (IC_50_) of common chemotherapeutic drugs was generated by the “pRRophetic” R package in different risk groups. Cisplatin, paclitaxel, docetaxel, doxorubicin, etoposide, gemcitabine, methotrexate, and cytarabine were included in this research.

### Cell culture and qRT-PCR

The human normal nasopharyngeal cell line (NP69), the human tongue squamous cell carcinoma cells (SCC25) and the human NPC cell line (HNE-2) were donated by the Cancer Research Institute of Central South University (China). Human hypopharyngeal squamous cell carcinoma cells (FaDu) were purchased from Suzhou Bei Na Chuanglian Biotechnology Co., Ltd. (China). The cell culture conditions and qRT-PCR were as described earlier [18]. The list of primer sequences is described in Additional file 2: Table S2.

### Statistical analysis

Statistical analysis was visualized using R software (version 4.2.0, https://www.r-project.org/). The significance of the differences in the expression of lncRNAs between tumour and normal cell lines was assessed by the Wilcoxon test. Generally, *P* < 0.05 was considered significant unless otherwise specified.

## Results

### Identification of cuproptosis-related lncRNAs and patients’ clinical data

**Fig. 1:**
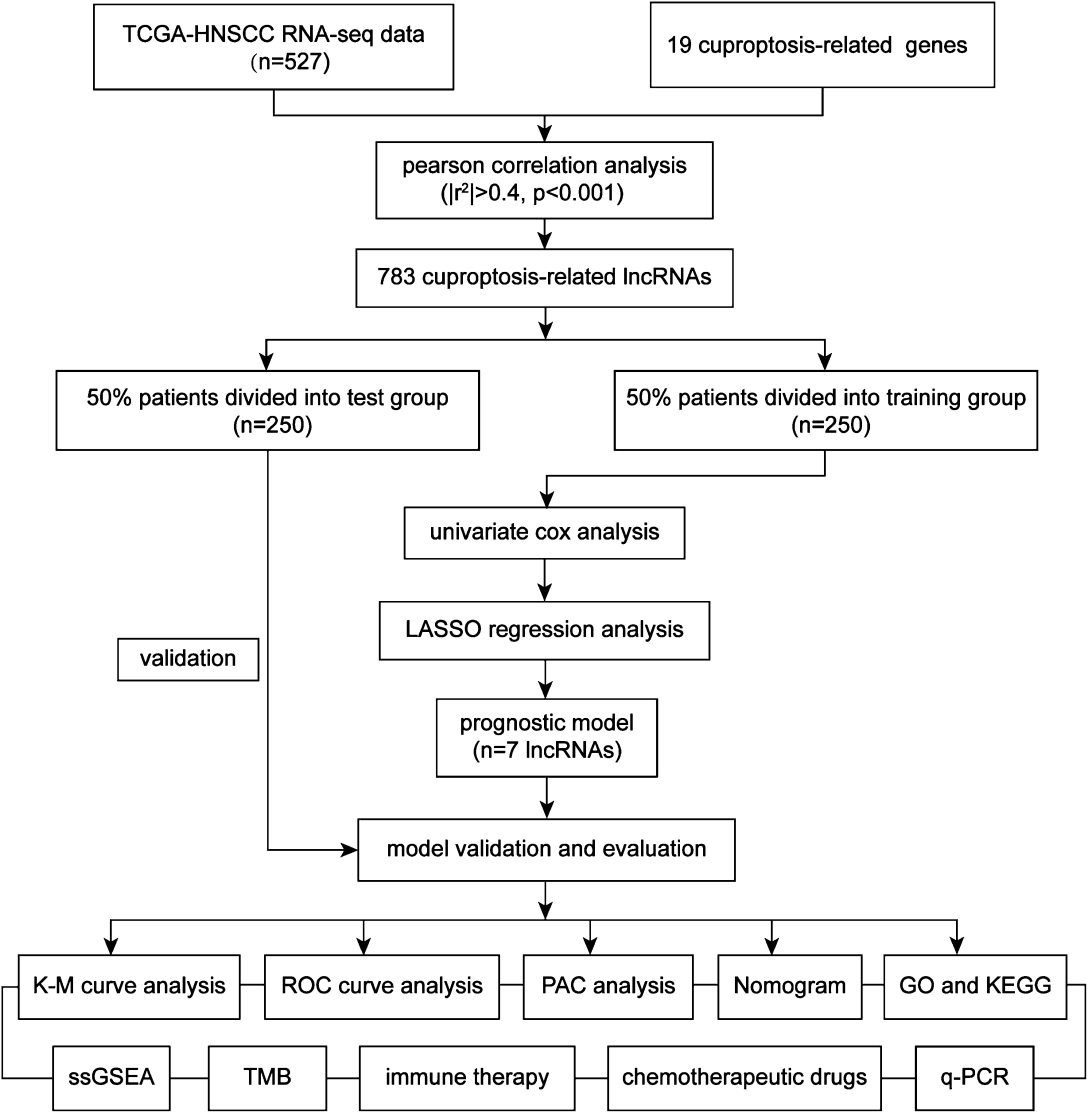
Flowchart of the construction and validation of the cuproptosis-related lncRNA signature

**Fig. 2:**
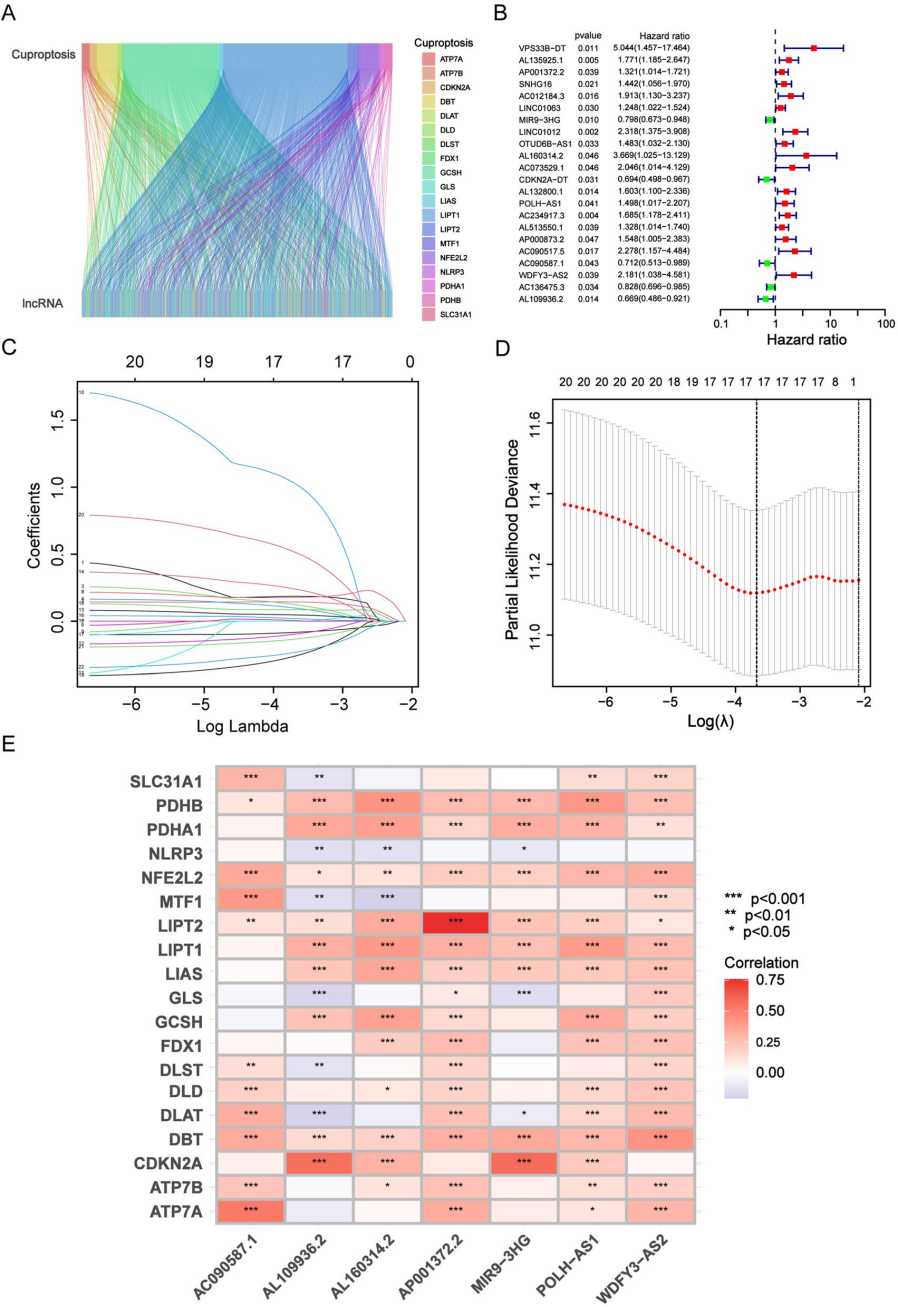
The coexpression analysis of the CRLs and CRGs and the analysis of independent prognostic potential. **a** The Sankey diagram presents the detail connection between CRLs and CRGs. **b** Forest plots shows the 22 prognostic CRLs. **c** LASSO coefficient profiles of 7 CRLs in HNSCC. **d** The optimal value of penalty lambda (λ) is selected in LASSO analysis. **e** The correlation between 7 prognostic CRLs and 19 CRGs in the TCGA-HNSCC cohort. The color of each unit shows the degree of correlation. *P < 0.05, **P < 0.01, and ***P < 0.001.

The flow chart is shown in Fig. 1. A total of 44 normal and 438 HNSCC samples with gene expression profiles and clinical data were obtained from the TCGA database, and the detailed clinical characteristics of the patients are shown in Table 1. After analysis, we identified 783 CRLs. Subsequently, a Sankey diagram was applied to visualize the internal connection between CRLs and CRGs (Fig. 2a). We also validated the prognostic potential of these CRLs based on Cox univariate regression analysis using OS data from the TCGA-HNSCC database. Ultimately, we identified 22 prognostic CRLs in HNSCC (Fig. 2b).

**Table 1:** The clinical characteristics of patients in the TCGA database

Variable	
Gender	
Male/female	385/142
Age at diagnosis	
≤ 65/>65/NA	345/181/1
Grade	
G1/G2/G3/G4/NA	63/311/124/7/22
Stage	
I/II/III/IV/NA	27/74/82/269/75
T	
T0/T1/T2/T3/T4/NA	1/49/140/101/174/62
M	
M0/M1/NA	190/1/330
N	
N0/N1/N2/N3/NA	179/68/172/8/100

### Construction and validation of a CRL prognostic model

To test the prognostic capability of these CRLs, we randomly divided the data from TCGA-HNSCC into a training group and a test group, and the detailed difference between the two groups was not significant (Additional file 3: Table S3). Then, from the aforementioned 22 prognostic CRLs of the training group, we used the optimal penalty parameter (λ) for the LASSO model to construct a prognostic risk assessment model based on only seven CRLs (Table 2). The cvfit curve and lambda curve are shown in Fig. 2c, d. In this signature, each patient with HNSCC in the TCGA database was assigned a risk score according to the following formula: Risk Score = AP001372.2 * 0.24342 + MIR9-3HG*(− 0.19975) + AL160314.2 * 1.46892 + POLH-AS1 * 0.51725 + AC090587.1 * (−0.41087) + WDFY3-AS2 * 0.96673 + AL109936.2 * (−0.50344). In addition, a heatmap was applied to exhibit the association between the 7-CRL-related prognostic model and 19 CRGs (Fig. 2E).

**Table 2:** Cox regression analysis with the LASSO algorithm for the prognostic model based on 7 CRLs

ID	Coefficient	HR	HR.95L	HR.95H	P-value
AP001372.2	0.24342464	1.32106993	1.01420121	1.72078848	0.03896856
MIR9-3HG	− 0.19974846	0.79831460	0.67257091	0.94756730	0.00999966
AL160314.2	1.46892173	3.66922945	1.02543466	13.12930542	0.04565404
POLH-AS1	0.51725214	1.49823901	1.01731396	2.20651660	0.04067063
AC090587.1	0.41086540	0.71193677	0.51254202	0.98890226	0.04271089
WDFY3-AS2	0.96673401	2.18108428	1.03837312	4.58132878	0.03945533
AL109936.2	0.50343907	0.66897369	0.48579348	0.92122644	0.01379458

**Fig. 3:**
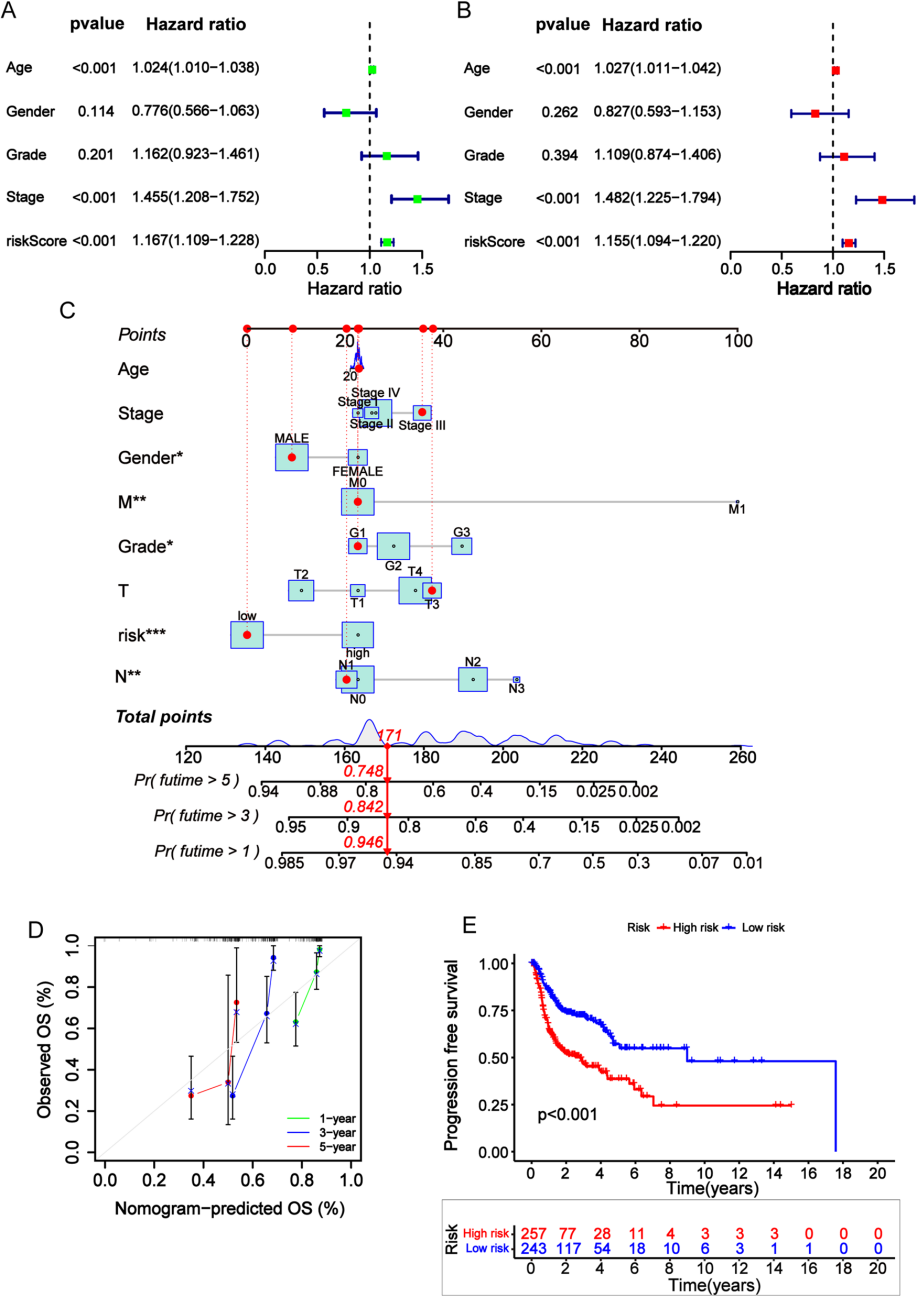
Prognostic analysis of the 7-CRL signature model in the TCGA cohort. **a, b** Results of the univariate Cox regression analysis and multivariate Cox regression analysis regarding OS of the 7-CRL signature. **c** Nomogram construction for predicting the 1-, 2- and 3- year OS of HNSCC patients. **d** The calibration curve of TCGA-HNSCC. The dashed diagonal line in grey color represents the ideal nomogram

Based on the univariate and multivariate Cox regression analysis revealed that the 7-CRL prognostic model, age, and stage could serve as independent prognostic factors for predicting the OS rates for HNSCC (Fig. 3a, b). The predicted nomogram calculates the probability of survival for those patients by summing the scores of many correlated factors in the scorecard, including risk score, age, clinical stage, sex, grade, and TNM stage. The calibration curve of the nomogram showed that there was good consistency between the predicted results and observed results. Compared with an ideal predictive model, it can accurately predict OS rates at 1, 3, and 5 years (Fig. 3c, d). The K–M survival curves demonstrated that HNSCC patients in the low-risk group had a significantly better PFS than those in the high-risk group (Fig. 3e). The results of PCA are shown in Additional file 4: Figs. S1–S4. The distribution of patients for all genes, cuproptosis-related genes, cuproptosis-related lncRNAs, and risk lncRNAs suggests that these lncRNAs can be reliably used to construct the signature.

**Fig. 4:**
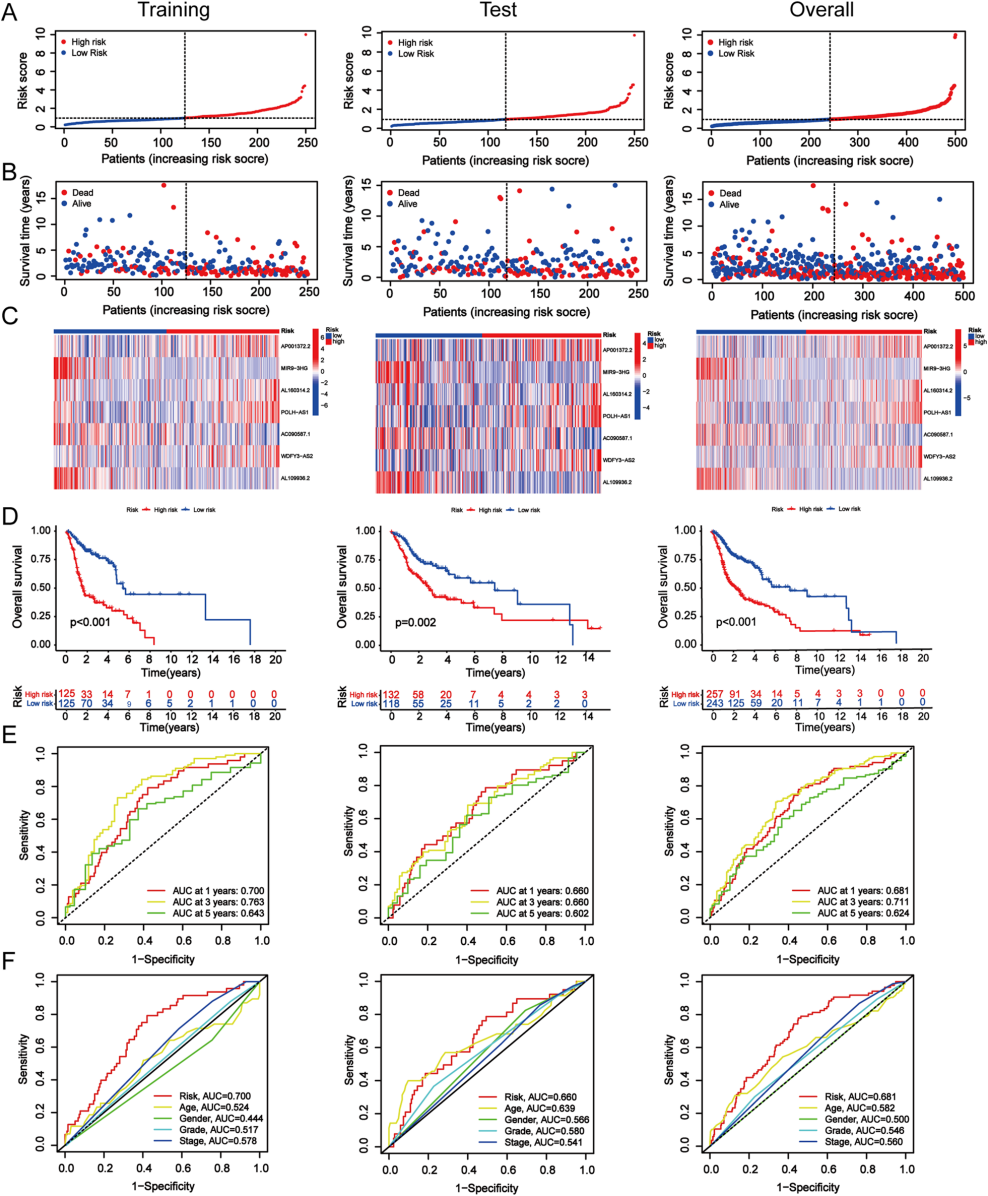
Validation of the CRL signature in the training, test and overall groups. **a, b** The distribution of the risk scores and OS status. **c** Prognostic signature signal heatmaps. **d** The K–M curves for survival status and survival time. **e** The ROC curve shows the potential of the prognostic CRL signature. **f** AUC curves comparing the prognostic accuracy of CRL signature

**Fig. 5:**
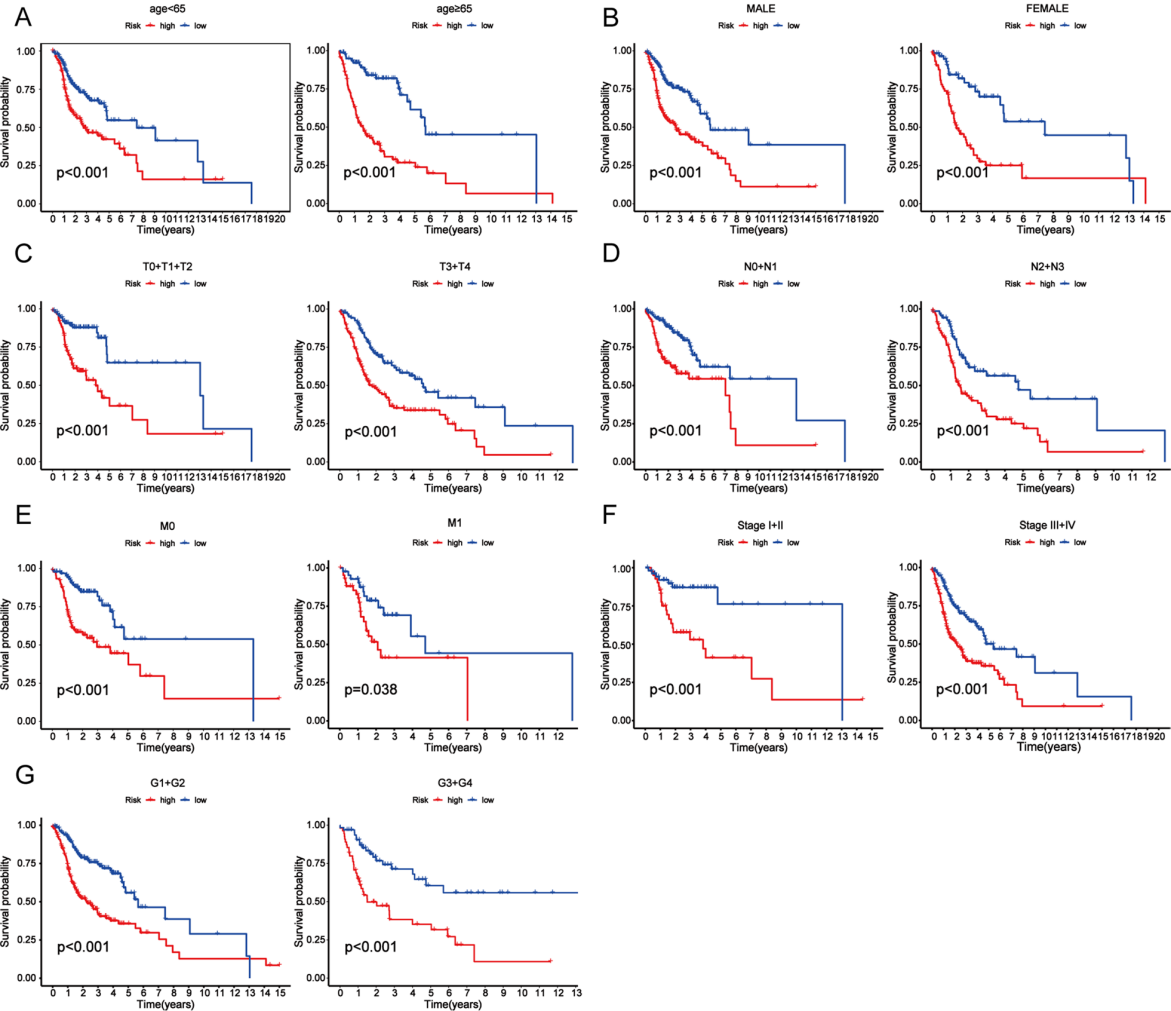
Subset group K–M survival curves of patients. **a** Age. **b** Gender. **c** T stage. **d** N stage. **e** M stage. **f** Stage. **g** G stage

Then, we estimated the prognostic performance of this 7-CRL model. The training group samples were then divided into high-risk and low-risk groups based on the median risk scores. Forest plots of univariate and multivariate Cox regression also identified that the cuproptosis-related lncRNA model is associated with overall survival. Visualizing the distribution of the risk scores and the OS status is presented to demonstrate that the sample distribution of those two risk groups was reasonable (Fig. 4a, b). A heatmap presented the expression of the 7 CRL signatures (Fig. 4c). The prognostic effectiveness of this model for HNSCC patient OS status was evaluated using K–M survival curve analysis. The overall survival rate of patients with HNSCC was worse in the high-risk group than that in the low-risk group (Fig. 4d). We also generated a time-related ROC curve in the training group. The areas under the curve (AUC) remained above 0.64 at the 1-year, 3-year, and 5-year points (Fig. 4e). The excellent prognostic accuracy of this model was validated by the ROC curve compared to other clinicopathological information (Fig. 4f). To further assess the predictive power of this 7-lncRNA signature, double validation was performed on distribution figures, heatmaps, K–M survival analysis and ROC analysis for the validation group and the overall group. Patients in the above two risk groups were also reasonably distributed in the test group and the overall group. All these findings suggest that the 7-CRL signature could serve as a valuable prognostic model for HNSCC. Moreover, the high-risk group presented higher mortality rates than the low-risk group. We also performed the K–M survival analysis among each subset group that was separated according to the clinicopathological features. The results are shown as subset groups separated by age, sex, T stage, N stage, M stage, clinical stage, and grade. All of the K–M survival analyses in different clinical subgroups also showed that the high-risk group had worse survival than the low-risk group (Fig. 5).

### Discovery of molecular functions and pathways by GO and KEGG analysis

**Fig. 6:**
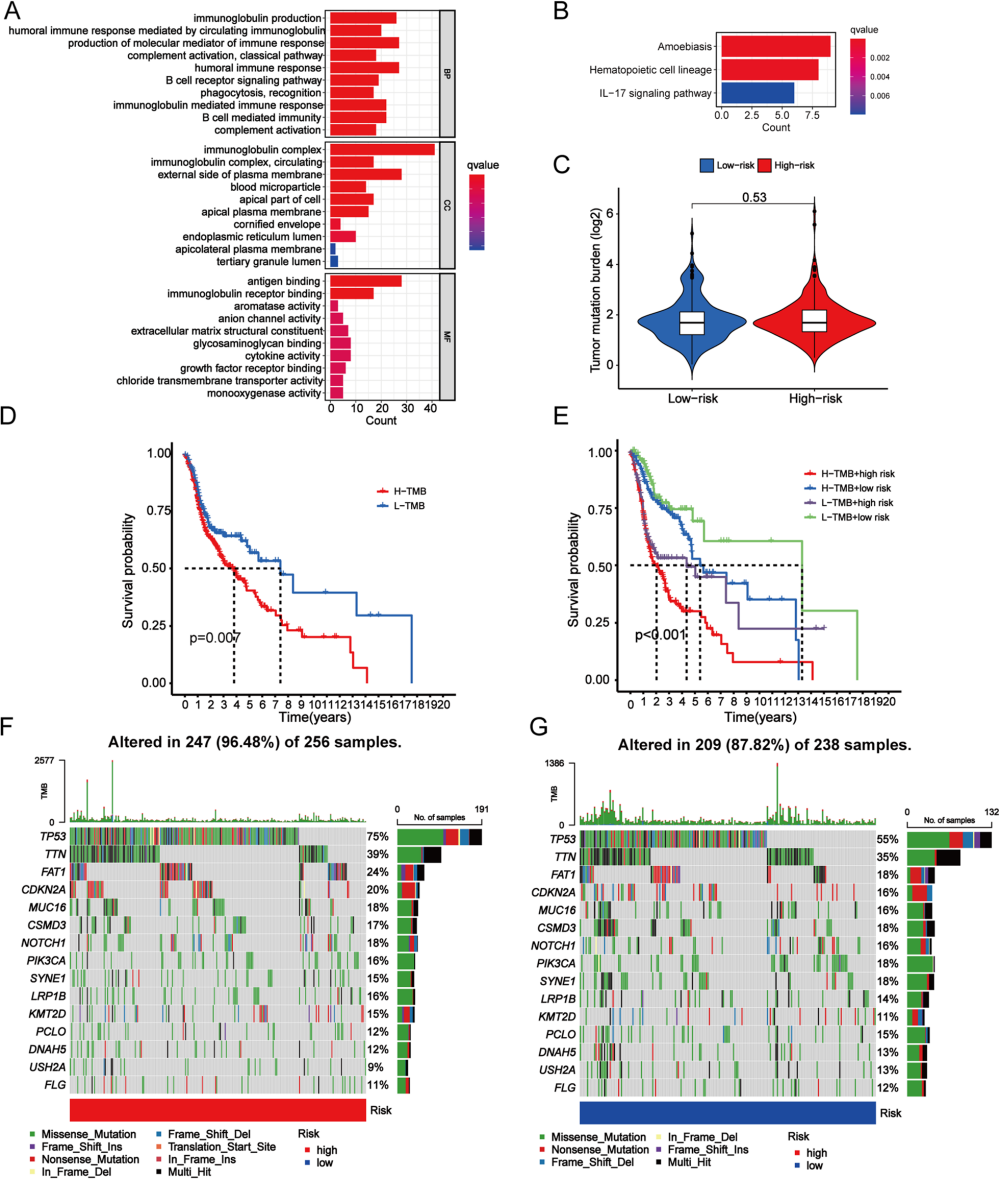
Enrichment analysis and TMB analysis based on the CRL prognostic signature in high- and low-risk HNSCC patients. **a, b** GO and KEGG analysis showing immune-related biological processes and pathway were enriched. **c** The TMB scores between high-risk and low-risk groups. **d** K–M survival analysis of the TMB score. **e** K–M survival analysis of the risk score combined with the TMB score. The waterfall graphs of most frequently mutated genes in high-risk group (**f**) and in low-risk group (**g**)

GO enrichment analysis showed that these DEGs were mainly enriched in immunoglobulin production, immunoglobulin-mediated immune response, immunoglobulin-mediated immune response, immunoglobulin complex, and antigen binding; KEGG pathway analysis showed that these DEGs were mainly enriched in amoebiasis, haematopoietic cell lineage, and IL-17 signalling pathway (Fig. 6a, b). In summary, these findings revealed that the 7-CRL signature was associated with tumour immunity in HNSCC.

### Tumour mutation burden (TMB)

By analysing the tumour mutation burden, no significant differences were found between the low-risk groups and high-risk groups of HNSCC patients (*P* > 0.05, Fig. 6c). Interestingly, it was observed that patients with higher TMB appeared to have worse prognosis, as shown in Fig. 6d. In particular, the combination of TMB score and risk score appears to have a better prognosis in patients with HNSCC. Patients with high TMB scores and high risk scores had the worst prognosis, while patients with low TMB scores and low risk scores had a better prognosis (Fig. 6e). It was also shown that genes with the most mutation frequencies between those two groups were different in the waterfall plot (Fig. 6f, g). We found that the high-risk group had a higher mutation frequency than the low-risk group (*P* < 0.05). TP53 (75 vs. 55%), TTN (39 vs. 35%), FAT1 (24 vs. 18%), CDKN2A (20 vs. 16%) and MUC16 (18 vs. 16%) had the top five mutation frequencies in the high-risk group and low-risk group. In conclusion, the TMB between the two groups was different, which might play a critical role in the development and progression of HNSCC.

### Comparison of immune functions and TIDE scores in different risk groups

**Fig. 7:**
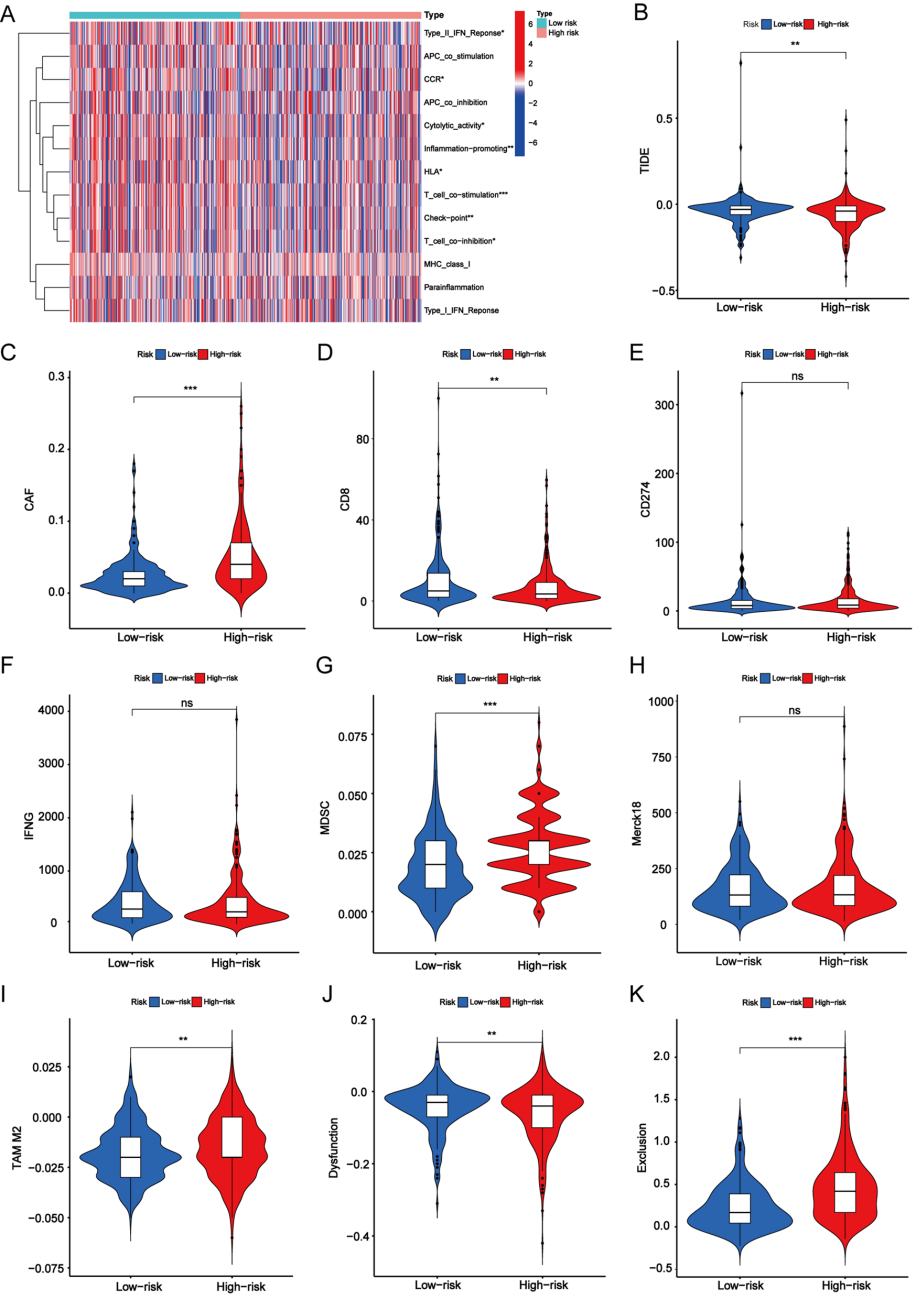
Immune related function in high-risk and low-risk groups. **a** Heatmap showing the distribution of immune function between high-risk and low-risk groups. TIDE score (**b**), CAF score (**c**), CD8 score (**d**), CD274 score (**e**), MDSC score (**g**), Merck18 score (**h**), TAM M2 score (**i**), T cells dysfunction score (**j**), T cells exclusion score (**k**) in high-risk and low-risk groups. *P < 0.05, **P < 0.01, and ***P < 0.001

We analysed immune-related functions to evaluate the immune status of the low-risk and high-risk groups, and the results showed that the type II interferon (IFN) response, cytolytic activity, inflammation promotion, T-cell co-stimulation, checkpoint, and T-cell co-inhibition were significantly more active in the low-risk group than in the high-risk group. In total, the immune functions in the low-risk group were higher than those in the high-risk group (Fig. 7a). We obtained the TIDE score, CAF score, CD8 score, CD274, IFNG score, MDSC score, merck18 score, TAM M2, T-cell dysfunction score, and T-cell exclusion score from the TIDE website. We found that the TIDE value of the low-risk group was higher than that of the high-risk group, suggesting that the high-risk group was less likely to escape the immune system and had a better response to immunotherapy (Fig. 7b). Then, we deeply investigated the distribution between those scores and risk groups, and the findings showed that the low-risk group had higher scores, except the CAF score, MDSC score, TAM M2 score, and T-cell exclusion score (*P* < 0.001) (Fig. 7c–k).

### Drug sensitivity in the 7-CRL signature

**Fig. 8:**
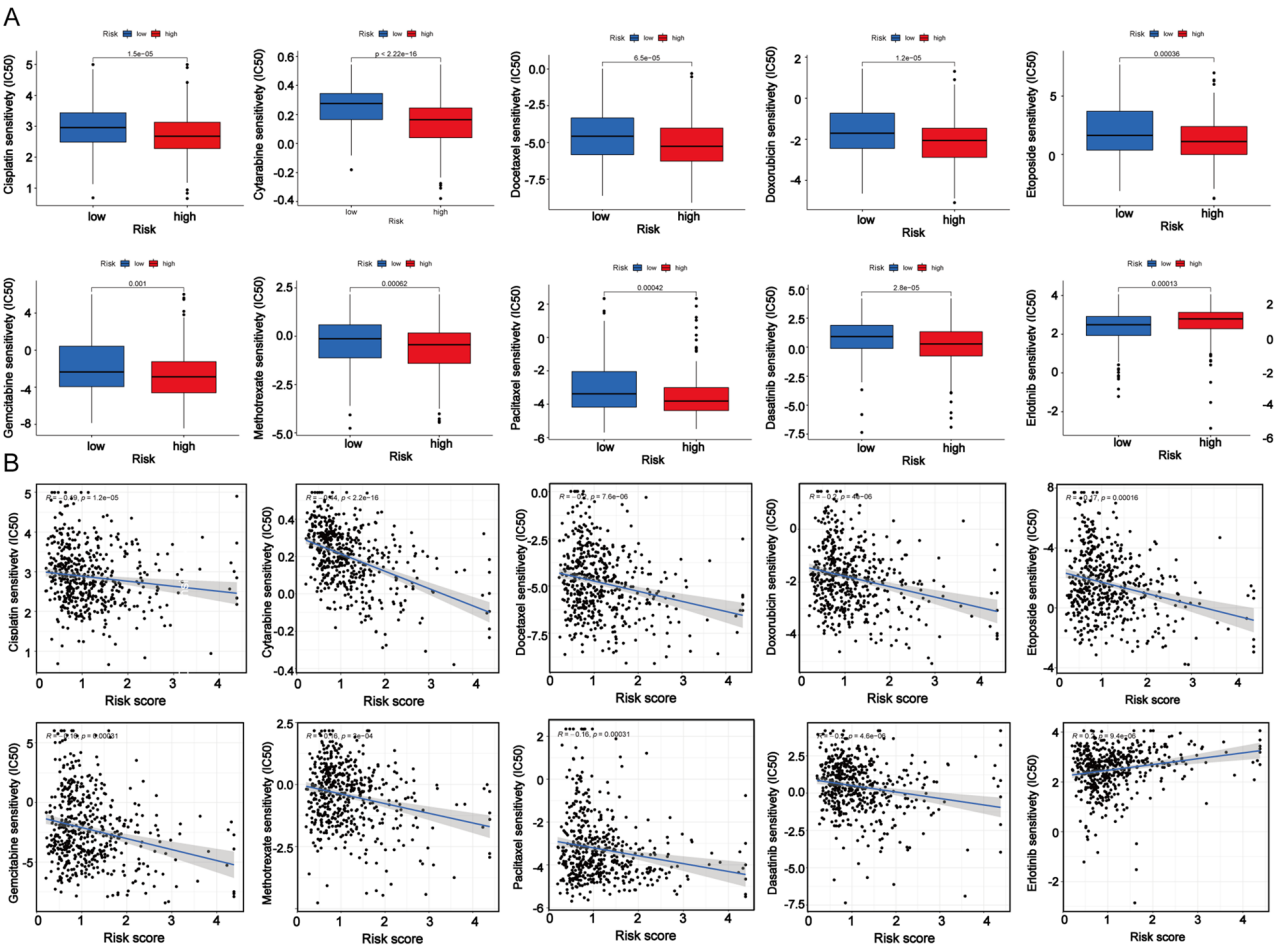
The analysis of drug sensitivity based on CRL signature in HNSCC. **a** Drug sensitivity (IC50) analysis of Cisplatin, Cytarabine, Docetaxel, Doxorubicin, Etoposide, Gemcitabine, Methotrexate, Paclitaxel, Dasatinib and Erlotinib. **b** The correlation of drug sensitivity (IC50) and cisplatin, Cytarabine, Docetaxel, Doxorubicin, Etoposide, Gemcitabine, Methotrexate, Paclitaxel, Dasatinib, Erlotinib

To further investigate the differences in the drug resistance potential in the two risk groups, we estimated and compared the IC_50_ values of drugs or inhibitors in those above groups. Then, 10 representative drugs are shown in Fig. 8. We found that cisplatin, cytarabine, docetaxel, doxorubicin, etoposide, gemcitabine, methotrexate, paclitaxel, and dasatinib may be predictive candidates for the treatment of patients in the high-risk group. Erlotinib may not act as an ideal drug for patients in the high-risk group. The risk score was negatively related to the IC_50_ values of all drugs except erlotinib.

### Validation of 7-CRL in HNSCC

**Fig. 9:**
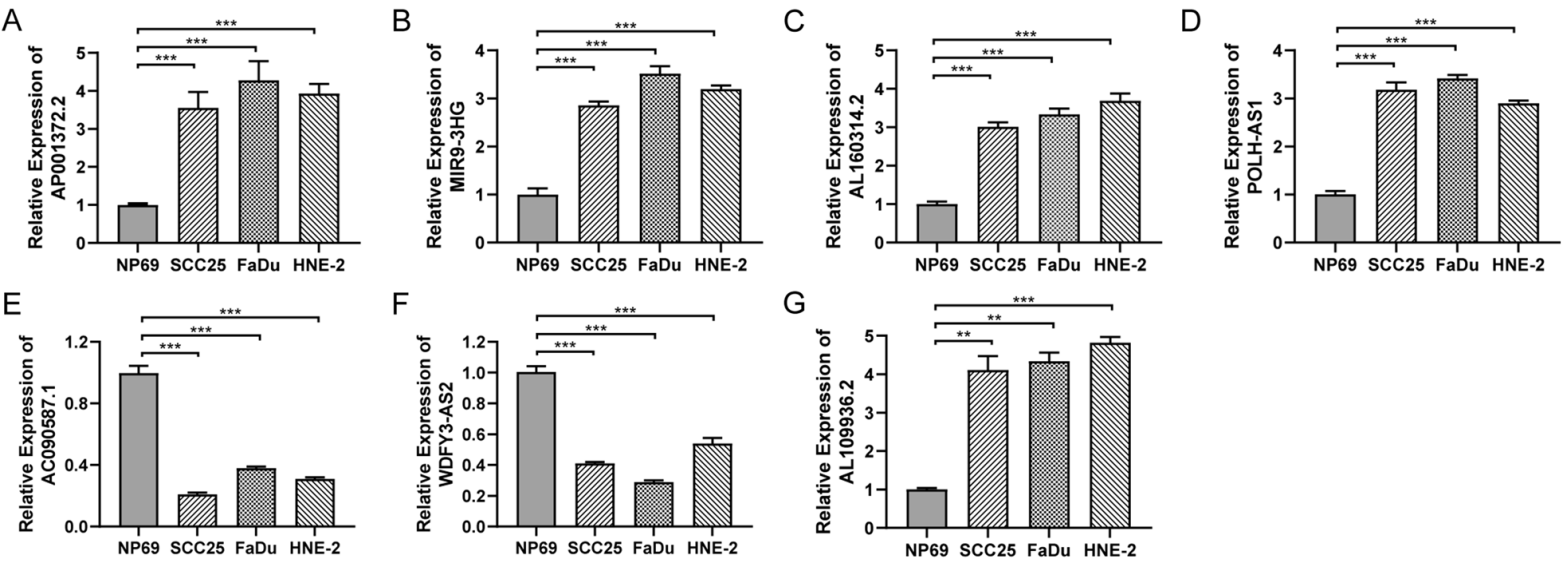
The expression of AP001372.2, MIR9-3HG, AL160314.2, POLH-AS1, AC090587.1, WDFY3-AS2 and AL109936.2 in nasopharyngeal normal cell lines and HNSCC cell lines cell lines by qPCR. *P < 0.05, **P < 0.01, and ***P < 0.001

To further explore the expression of these seven prognostic CRLs, qRT-PCR analysis showed that the expression levels of AP001372.2, MIR9-3HG, AL160314.2, POLH-AS1, and AL109936.2 were upregulated, while AC090587.1 and WDFY3-AS2 were downregulated in HNSCC cell lines compared with normal cell lines (Fig. 9). Moreover, these findings showed that AP001372.2, MIR9-3HG, AL160314.2, POLH-AS1, AC090587.1, WDFY3-AS2, and AL109936.2 may play an important role in development and progression of HNSCC.

## Discussion

As a novel form of programmed cell death, cuproptosis is a type of copper-dependent cell death that is distinct from apoptosis, necroptosis, and ferroptosis [19]. Ferroptosis is triggered by the iron-dependent peroxidation of oxidizable membrane phospholipids [20]. Similar to ferroptosis, cuproptosis derives from excessive intracellular copper-induced accumulation of lipoylated dihydrolipoamide S-acetyltransferase (DLAT), which is related to the mitochondrial tricarboxylic acid (TCA) cycle; finally, it results in proteotoxic stress and the occurrence of cell death [12]. Moreover, Tsvetkov et al. further identified a series of key genes or proteins involved in the process of cuproptosis, which was closely associated with copper imbalance [12]. Several studies have indicated that copper homeostasis plays an important role in the development of HNSCC and that regulating copper metabolism affects the cell growth and proliferation of different cancers [21–23]. To the best of our knowledge, this was the first study to investigate the possible roles and mechanisms of cuproptosis in the development, prognosis, and chemotherapy efficacy of HNSCC.

In the present study, we collected and obtained crucial genes related to cuproptosis from previous studies to identify the cuproptosis-related lncRNAs that were candidates for the prognostic signature for HNSCC. Then, a novel prognostic 7-lncRNA model was constructed that was significantly correlated with the prognosis of HNSCC. In previous investigations, several ferroptosis-related signatures were developed to predict the prognosis of HNSCC patients [24]. Some studies have also shown that prognostic cuproptosis-related signatures have been established for various tumours, including osteosarcoma, lung adenocarcinoma, and liver cancer [25–27]. However, the prognostic signature of cuproptosis-related lncRNAs in HNSCC has rarely been explored. Here, we reported that the seven CRLs were differentially expressed between tumour and normal cell lines and were associated with the OS and PFS of HNSCC, suggesting a potential role in the prediction of HNSCC survival. Additionally, we found that the expression of AP001372.2, MIR9-3HG, AL160314.2, POLH-AS1, and AL109936.2 was upregulated and AC090587.1 and WDFY3-AS2 were downregulated in tumour cell lines compared with normal cell lines. The detailed mechanisms of seven cuproptosis-related lncRNAs in cancer progression and prognosis are unknown. Hence, despite the important prognostic value of the cuproptosis-related lncRNA signature identified in this study, future research is urgently needed to elucidate their mechanisms in HNSCC.

In this study, the low-risk groups presented higher TIDE scores. However, a higher TIDE score has been reported to be associated with lower responsiveness to both anti-PD-1 and anti-CTLA-4 treatment [28]. Due to our results, no significant differences in CD274 (PD-L1) or TMB score were observed between the two risk groups. However, several studies have suggested that high TMB may serve as a preferable biomarker, which optimizes the efficacy of PD-1/PD-L1 inhibition in improving survival, and the Food and Drug Administration (FDA) has approved pembrolizumab for the treatment of solid tumours with high TMB (TMB-H or TMB ≥ 10) [17, 29, 30]. Interestingly, patients with lower TMB scores and lower risk scores had a better prognosis in the long term. Therefore, in our opinion, in contrast to other solid cancers, the immunotherapy efficacy and prognosis of HNSCC might be determined by a combination of multiple factors, including the TIDE score, PD-L1 expression, TMB scores, and risk scores.

Of the seven CRLs in our study, MIR9-3HG, POLH-AS1, and WDFY3-AS2 are reported to play important roles in various cancers by regulating ferroptosis [31–33]. Additionally, POLH-AS1 was identified to regulate the process of necroptosis in hepatocellular carcinoma [34]. It was also reported that MIR9-3HG knockdown inhibited cell proliferation and promoted apoptosis in cervical cancer [35]. Silencing WDFY3-AS2 significantly inhibited proliferation, migration and invasion but accelerated cell apoptosis in cisplatin-resistant ovarian cancer. Obviously, these three lncRNAs were involved in a series of programmed cell death pathways, including cuproptosis, ferroptosis, apoptosis, and necroptosis, indicating that the different types of programmed cell death could be seen as a single, coordinated cell death system in which the individual pathways are highly interconnected and can flexibly compensate for one another. Hence, despite the important prognostic value of the CRL signature identified in our study, future studies are needed to elucidate their mechanisms in HNSCC.

There were some limitations in our study. First, we only performed internal verification based on the TCGA database, and we still need other databases for external validation of our signature. Second, the mechanism of CRLs in HNSCC remains to be further identified in HNSCC tissues compared with control tissues.

## Conclusions

A novel CRL signature was constructed for predicting the chemotherapy efficacy and prognosis of HNSCC. This 7-CRL prognostic model deserves further attention.

## Supplementary Information

**Additional file 1: Table S1.** The list of 19 CRGs.

**Additional file 2: Table S2.** The list of primer sequences of 7 CRLs.

**Additional file 3: Table S3.** No significant differences were observed in clinicopathological characteristics among training group, test group and overall group.

**Additional file 4: Table S4.** Threshold cycle (Ct) values from qRT-PCR of HNSCC cell lines and human normal nasopharyngeal cell line.

**Additional file 5: Figs. S1–S4.** The principal component analysis between low-risk and high-risk groups based on the expression of all genes (S1), cuproptosis-related genes (S2), lncRNAs (S3) and 7 CRLs (S4).

## Data Availability

The datasets supporting the conclusions of this article are included within the article and its additional files.

## Abbreviations

TCGA: The Cancer Genome Atlas
K–M: Kaplan–Meier
ROC: Receiving operating characteristic
GO: Gene Ontology
KEGG: Kyoto Encyclopedia of Genes and Genomes
LASSO: Least absolute shrinkage and selection operator
HNSCC: Head and neck squamous cell carcinoma
TMB: Tumor Mutation Burden
ssGSEA: Single-sample gene set enrichment analysis
PCA: Principal coordinate analysis
OS: Overall survival
AUC: Areas under the curve
q-PCR: Quantitative real-time polymerase chain reaction
TIDE: Tumor immune dysfunction and exclusion
CAF: Cancer-associated fibroblast
MDSC: Myeloid-derived suppressor cell
TAM: Tumor-associated macrophages
IC50: Half-maximal inhibitory concentration
