# Supplementary material for: Identification and validation of cuproptosis-related LncRNA signatures as a novel prognostic model for head and neck squamous cell cancer
Source: Cancer Cell Int. 2022 Nov 11;22:345. doi: 10.1186/s12935-022-02762-0 (PMC9652850; doi:10.1186/s12935-022-02762-0)
Supplement: Supplementary file 4 — Additional file 4: Table S4. Threshold cycle (Ct) values from qRT-PCR of HNSCC cell lines and human normal nasopharyngeal cell line. [file 12935_2022_2762_MOESM4_ESM.docx]

Table S4 Threshold cycle (Ct) values from qRT-PCR of HNSCC cell lines and human normal nasopharyngeal cell line

|  | AP001372.2 | | MIR9-3HG | | AL160314.2 | | POLH-AS1 | | AC090587.1 | | WDFY3-AS2 | | AL109936.2 | |
| --- | --- | --- | --- | --- | --- | --- | --- | --- | --- | --- | --- | --- | --- | --- |
|  | Gene Ct | GAPDH Ct | Gene Ct | GAPDH Ct | Gene Ct | GAPDH Ct | Gene Ct | GAPDH Ct | Gene Ct | GAPDH Ct | Gene Ct | GAPDH Ct | Gene Ct | GAPDH Ct |
| NP69 | 29.65 | 15.06 | 28.16 | 17.22 | 28.38 | 15.33 | 28.01 | 16.85 | 25.4 | 17.05 | 26.34 | 16.85 | 27.34 | 15.85 |
|  | 29.59 | 15.01 | 28.53 | 17.56 | 28.21 | 15.59 | 27.96 | 16.89 | 25.37 | 17.19 | 26.24 | 16.89 | 27.24 | 15.75 |
|  | 29.55 | 15.08 | 28.24 | 17.38 | 28.24 | 15.42 | 28.15 | 16.77 | 25.27 | 16.98 | 26.25 | 16.98 | 27.25 | 15.58 |
| SCC25 | 28.45 | 15.46 | 25.46 | 15.92 | 29.07 | 17.84 | 25.4 | 15.74 | 26.8 | 16.34 | 26.74 | 16.14 | 28.25 | 18.92 |
|  | 28.21 | 15.61 | 25.41 | 16.09 | 29.18 | 17.76 | 25.37 | 15.88 | 26.67 | 16.18 | 26.72 | 16.05 | 28.43 | 18.84 |
|  | 28.11 | 15.52 | 25.49 | 16.14 | 29.14 | 18.07 | 25.27 | 15.82 | 26.72 | 16.07 | 26.68 | 15.98 | 28.49 | 18.87 |
| FaDu | 29.18 | 16.42 | 26.41 | 17.21 | 27.28 | 16.17 | 27.05 | 17.46 | 24.75 | 15.07 | 28.73 | 17.67 | 26.12 | 16.57 |
|  | 28.95 | 16.64 | 26.34 | 17.26 | 27.22 | 16.11 | 27.11 | 17.89 | 24.8 | 15.13 | 28.81 | 17.63 | 25.99 | 16.60 |
|  | 28.84 | 16.54 | 26.28 | 17.25 | 27.35 | 16.29 | 27.06 | 17.58 | 24.73 | 15.08 | 28.74 | 17.51 | 26.11 | 16.75 |
| HNE-2 | 28.44 | 15.96 | 27.67 | 18.53 | 26.33 | 15.35 | 25.43 | 15.73 | 28.23 | 18.43 | 27.47 | 17.04 | 25.61 | 16.48 |
|  | 28.31 | 15.61 | 27.74 | 18.39 | 26.45 | 15.49 | 25.38 | 15.65 | 28.18 | 18.25 | 27.41 | 17.19 | 25.60 | 16.36 |
|  | 28.26 | 15.72 | 27.69 | 18.46 | 26.32 | 15.42 | 25.42 | 15.85 | 28.28 | 18.15 | 27.27 | 17.12 | 25.68 | 16.21 |
