# Supplementary material for: Identification and validation of cuproptosis-related LncRNA signatures as a novel prognostic model for head and neck squamous cell cancer
Source: Cancer Cell Int. 2022 Nov 11;22:345. doi: 10.1186/s12935-022-02762-0 (PMC9652850; doi:10.1186/s12935-022-02762-0)

Fig. S1 The principal component analysis between low-risk and high-risk groups based on the expression of all genes.


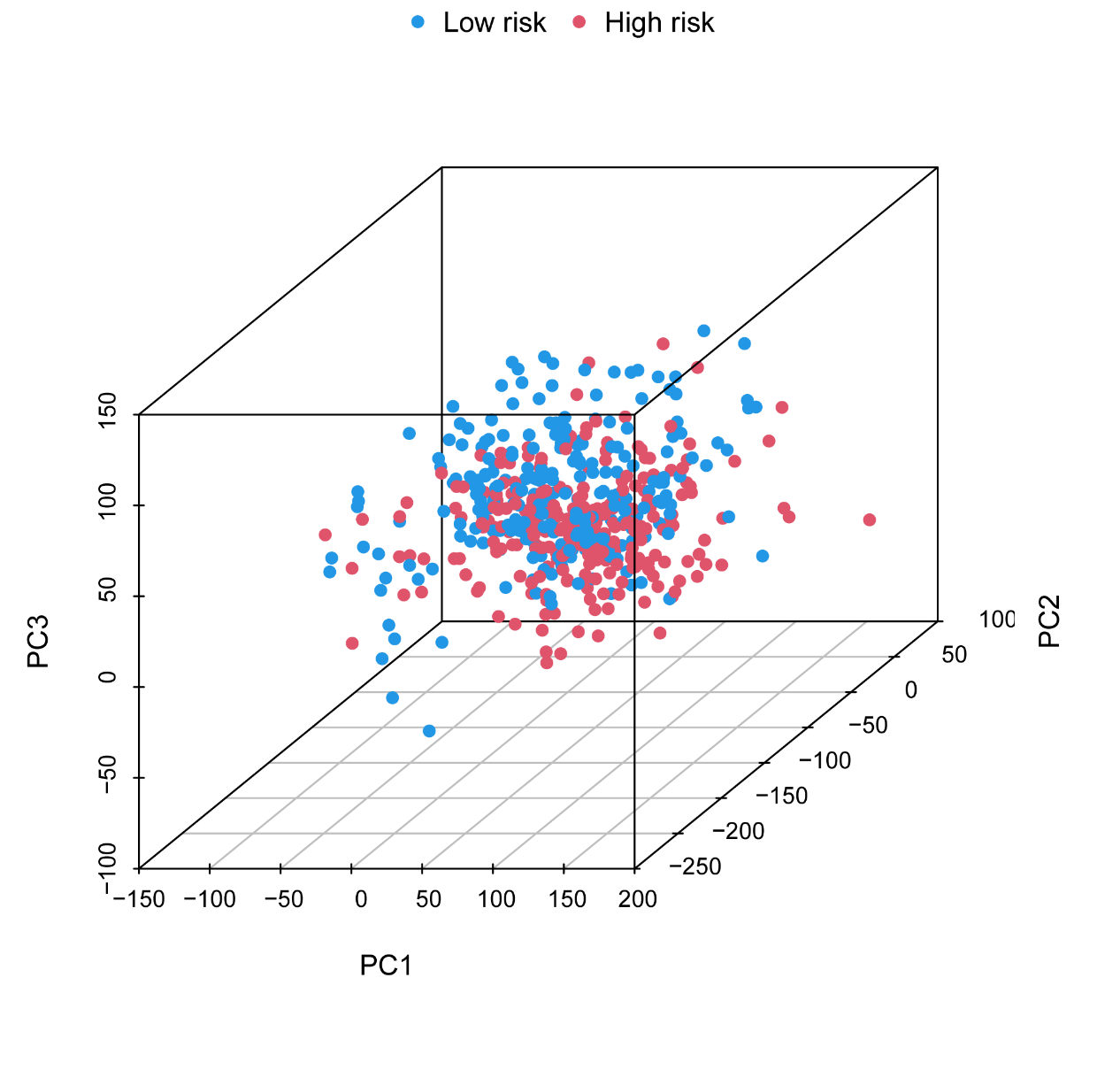


Fig. S2 The principal component analysis between low-risk and high-risk groups based on the expression of cuproptosis-related genes.


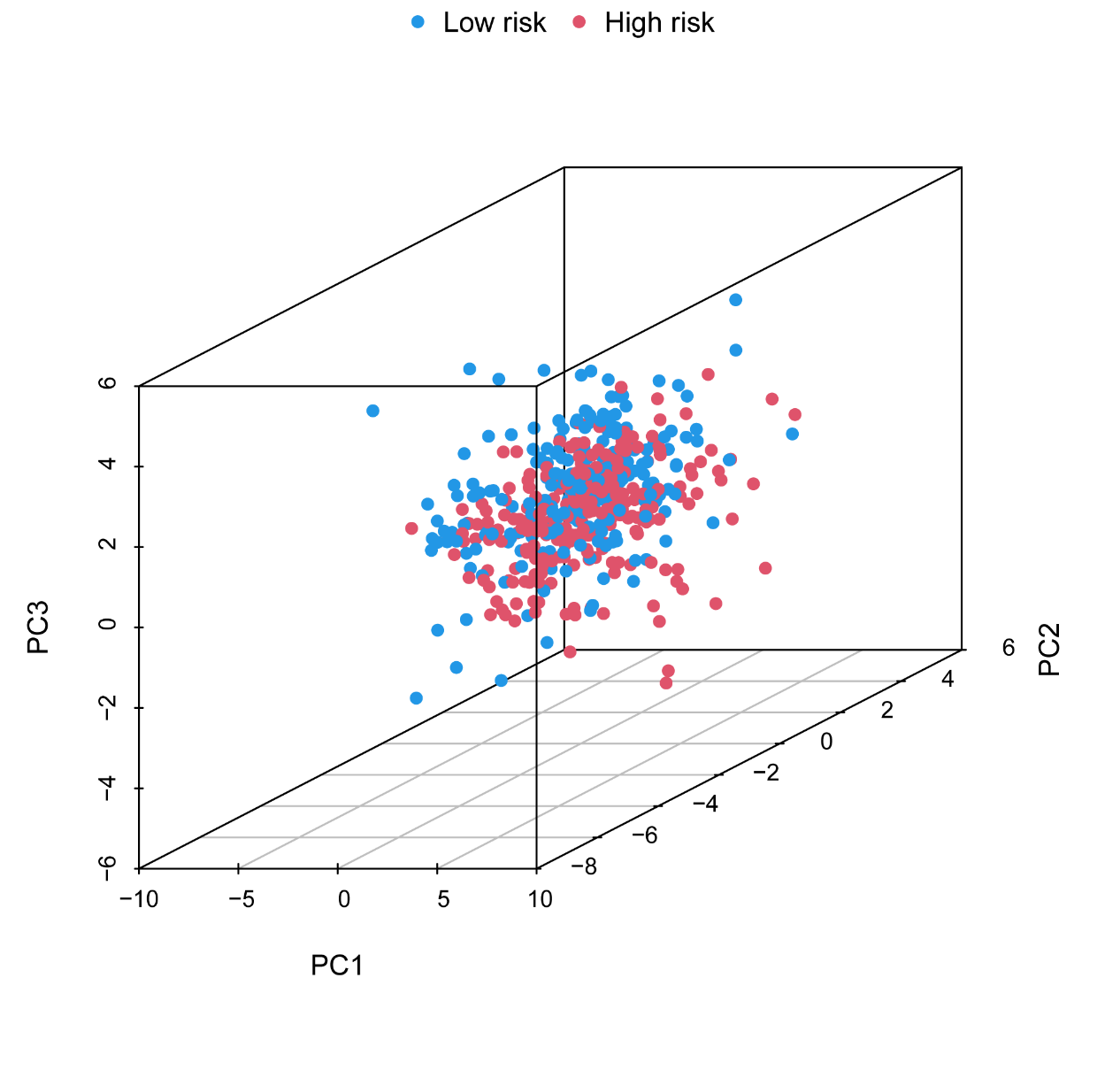


Fig. S3 The principal component analysis between low-risk and high-risk groups based on the expression of lncRNAs.


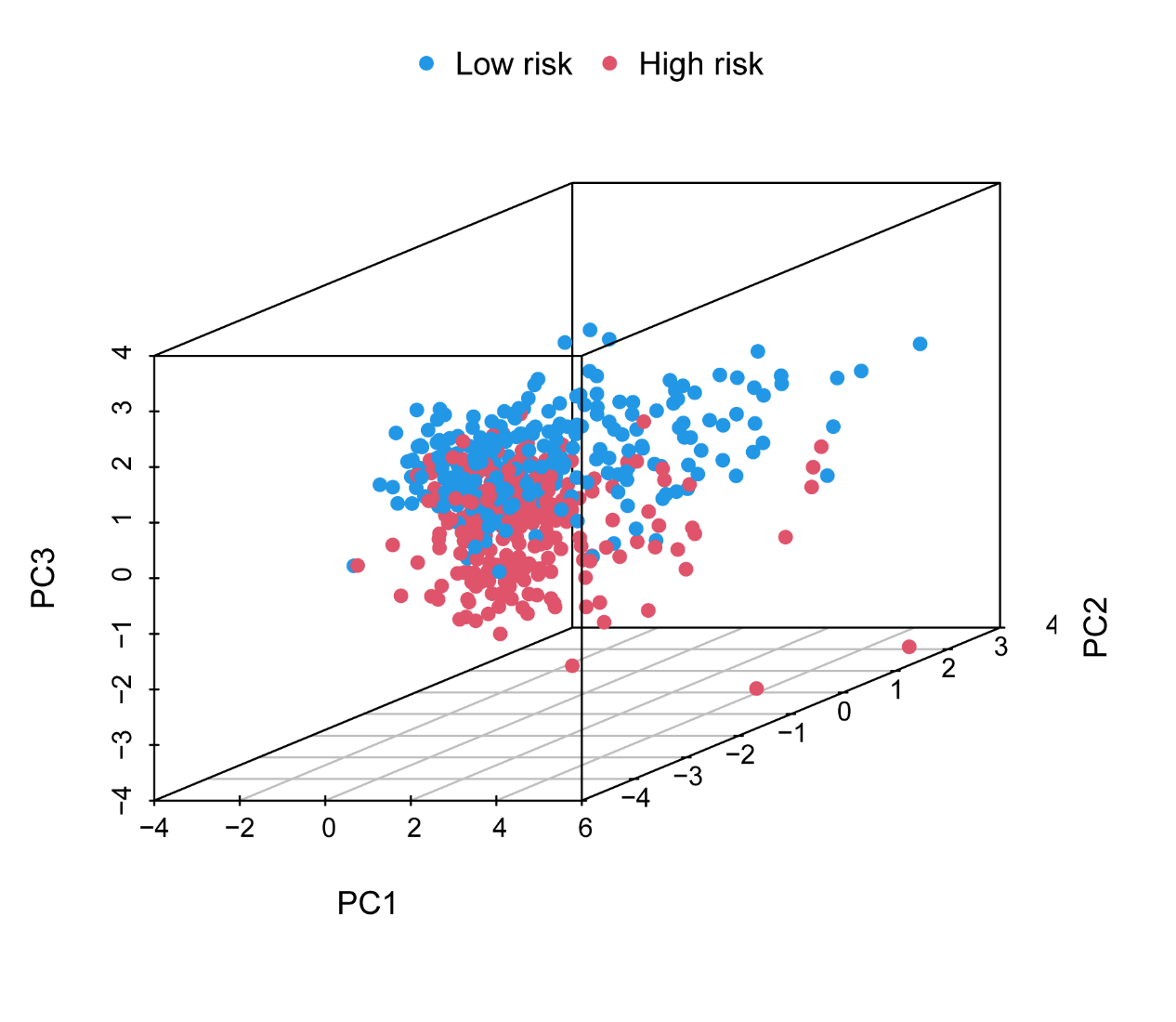


Fig. S4 The principal component analysis between low-risk and high-risk groups based on the expression of the 7 lncRNAs of the prognostic model.


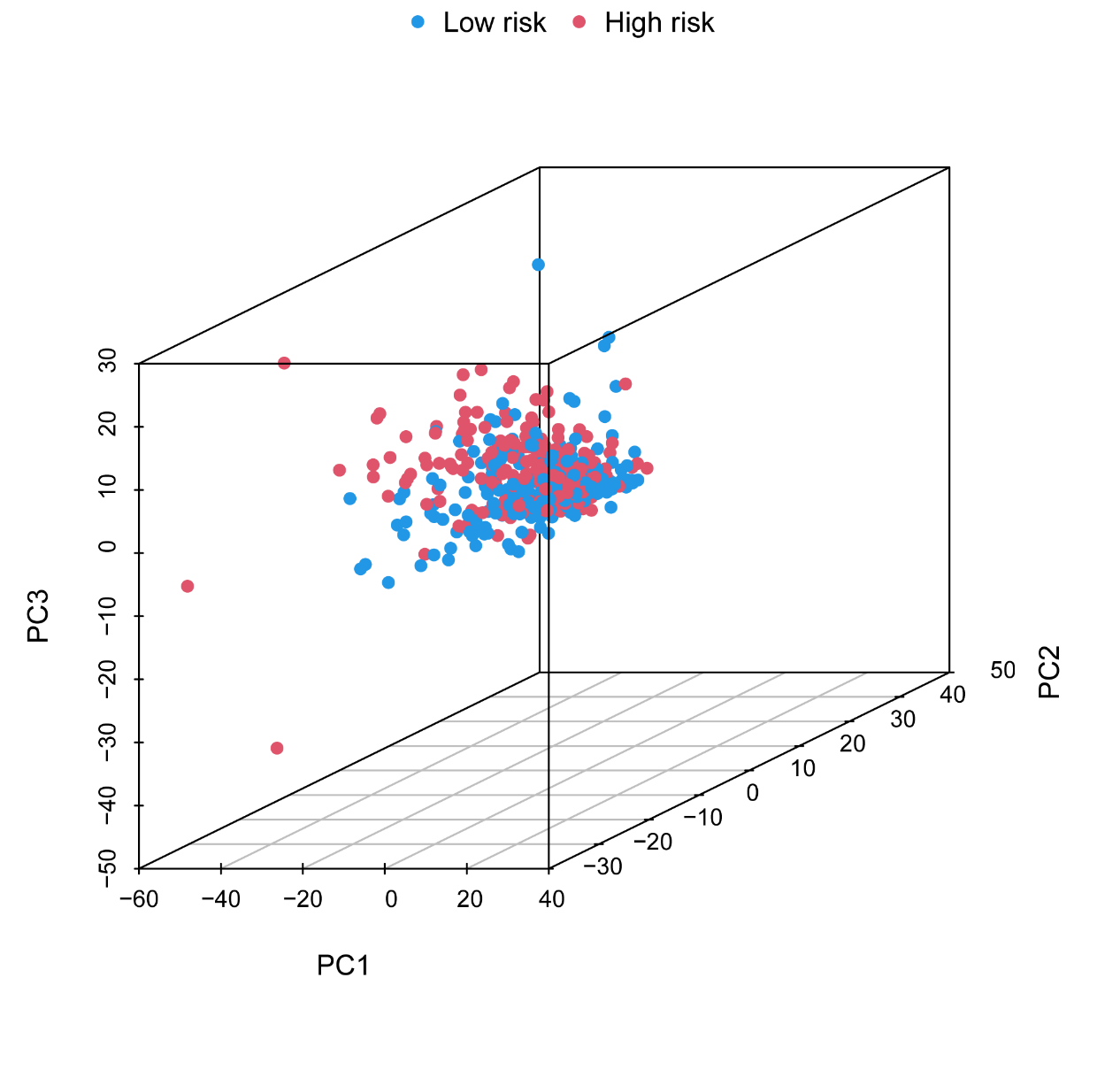

Supplement: Supplementary file 5 — Additional file 5: Figs. S1–S4. The principal component analysis between low-risk and high-risk groups based on the expression of all genes (S1), cuproptosis-related genes (S2), lncRNAs (S3) and 7 CRLs (S4). [file 12935_2022_2762_MOESM5_ESM.docx]
